# Efficacy of posterior teeth mesialization with invisalign in moderate anchorage cases

**DOI:** 10.1186/s12903-025-07518-6

**Published:** 2025-12-30

**Authors:** Yuanyou Lin, Rui Ma, Zhijie Luo, Lina Yan, Hongling Zhou, Lixing Zhao

**Affiliations:** 1 State Key Laboratory of Oral Diseases & National Center for Stomatology & National Clinical Research Center for Oral Diseases & Department of Orthodontics, West China Hospital of Stomatology, Sichuan University, No. 14, Third Section, Renminnan Rd, Chengdu, 610041 China; 2 Center of Stomatology, West China Xiamen Hospital of Sichuan University, Xiamen, Fujian 361021 China

**Keywords:** Clear aligners, Tooth movement, Mesial tipping, Moderate anchorage, Attachment design

## Abstract

**Background:**

Clear aligner therapy has become increasingly common for extraction cases, yet achieving controlled bodily mesialization of posterior teeth remains challenging, particularly in moderate anchorage situations. Clinical experience suggests that aligners often cause molar tipping and loss of anchorage, but few studies have quantified this problem under realistic clinical conditions. This study aimed to evaluate the predictability of posterior tooth movement with Invisalign in moderate anchorage cases and to compare the performance of different attachment designs.

**Methods:**

This retrospective study analyzed 32 patients (24 females, 8 males; mean age 23.9 ± 6.4 years) treated with bilateral first premolar extractions and Invisalign aligners. Digital dental models before and after treatment were superimposed using a surface-based registration protocol to assess tooth movement in three dimensions. The designed and achieved displacements, inclinations, rotations, and tipping angles of posterior teeth were compared using paired t-tests and one-way ANOVA.

**Results:**

Significant differences were observed between predicted and achieved values for mesial displacement and tipping in most posterior teeth. Maxillary first molars showed greater mesial movement and tipping than planned, while mandibular molars exhibited less movement but still notable tipping. Among attachment designs, the double attachment provided superior control of molar tipping compared to power arm and single attachment. Vertical skeletal pattern significantly influenced the expression of tipping and buccolingual inclination.

**Conclusion:**

In moderate anchorage extraction cases, posterior mesialization with clear aligners showed limited predictability, particularly due to molar mesial tipping. The use of double rectangular attachments appeared to provide better control of crown angulation than single attachment or power arm. These findings may help inform digital treatment planning and attachment selection for extraction cases requiring controlled posterior movement.

## Introduction

With the ongoing advancement of clear aligner therapy and the growing emphasis on comfort and facial esthetics, clear aligners have become increasingly favored by both patients and orthodontists [1, 2] Their appeal lies in advantages such as improved comfort, enhanced oral hygiene, removability, and superior esthetics [3–6]. In recent years, the integration of biomechanical principles from the fixed appliance into clear aligner protocols has further improved treatment efficiency and broadened the scope of clinical indications [7]. Clear aligners are now used in addressing protrusive facial profiles under maximum anchorage, resolving crowding in non-extraction cases, and achieving molar distalization with the aid of temporary anchorage devices [8–10]. In some clinical scenarios, outcomes with clear aligners have even surpassed those achieved with traditional fixed appliances [11].

Despite these advancements, challenges persist particularly in extraction cases that require bodily posterior tooth movement. In moderate anchorage situations, typically defined as cases requiring ≥ 2 mm of mesial movement of the posterior teeth, clinicians often encounter complications such as aligner disengagement and mesial tipping of molars. These side effects not only compromise the predictability and efficiency of space closure but may necessitate the use of additional mechanics such as segmented archwire techniques, thereby undermining the foundational principle of truly bracket-free orthodontics. As such, improving anchorage control and ensuring bodily movement of posterior teeth with clear aligners, while minimizing undesirable tipping, remains a critical issue in current orthodontic research.

Anchorage control remains a fundamental determinant of treatment success in extraction cases. Compared with Caucasian populations, Asian patients typically present with more protrusive dentofacial profiles, making premolar extraction a more commonly adopted approach [12]. In maximum anchorage scenarios, clear aligner therapy combined with implant anchorage can provide satisfactory posterior control. However, in moderate anchorage cases, where mesial movement of the posterior dentition is required, unintended tipping and loss of bodily control are frequently observed and are difficult to fully correct using aligners alone. Various adjunctive strategies, such as the incorporation of power arm, dual attachments and overcorrection, have been proposed, but none have reliably prevented mesial tipping of molars [13–15].

Previous studies [16, 17] have suggested that, in first premolar extraction cases, space closure achieved with clear aligners is generally less efficient than with fixed appliances, largely due to limitations in anchorage management. While the Invisalign system allows clinicians to digitally program detailed tooth movements, including molar mesialization, anterior retraction, molar angulation, and incisor torque, actual tooth movements often deviate from the predicted outcomes [18] Numerous studies [19–21] have documented excessive mesial movement and mesial tipping of the first molars during the retraction phase, underscoring persistent challenges in achieving effective anchorage control with aligner therapy.

Li et al. [22] reported that appropriate attachment design may contribute to improved root angulation during space closure. However, Dai et al. [21] found no significant difference in controlling mesial tipping of maxillary first molars when comparing horizontal and vertical rectangular attachments. Moreover, Vongtiang et al [13] demonstrated that in extraction cases requiring maximum anchorage, the use of power arms caused significantly greater mesial displacement and tipping of the first molars compared to the control group. These findings collectively suggest that current auxiliary devices remain insufficient in fully preventing mesial tipping of posterior teeth during space closure.

This research focused on posterior tooth mesialization with clear aligners under moderate anchorage conditions, in which controlled anterior retraction and posterior advancement are both required for space closure. This clinical scenario contrasts with previous investigations that concentrated on maximum anchorage protocols, where the posterior teeth were primarily maintained in position. Therefore, the present study aimed to evaluate the accuracy of posterior tooth mesialization with clear aligners under moderate anchorage conditions and to examine how different factors (age, sex, and vertical skeletal pattern) affect the expression of planned tooth movement. Furthermore, the study compared the effectiveness of various attachment designs in controlling mesial tipping of the first molars, with the ultimate goal of reducing unwanted mesial tipping and improving the predictability of bodily movement.

## Materials and methods

The study protocol was reviewed and approved by the Institutional Review Board of No. WCHSIRB-D-2025-381.

### Study design and participants

This retrospective study included 32 patients (24 females, 8 males; mean age 23.9 ± 6.4 years) who received clear aligner treatment with bilateral first premolar extractions at the Department of Orthodontics, West China Hospital of Stomatology, Sichuan University, between January 2020 and December 2024. All participants completed the first series of aligners without midcourse refinement.

Given the purpose of this study, a clear operational definition of anchorage level was necessary to ensure consistent sample selection. Moderate anchorage was defined according to the classical orthodontic classification in which posterior teeth are allowed to move mesially into approximately one-quarter to one-half of the extraction space during space closure [23–25]. This proportion corresponds to cases involving reciprocal anterior retraction and posterior mesialization, rather than stationary posterior segments observed in maximum anchorage control. Considering that the average first-premolar extraction space measures about 7 mm [23], this range approximately equals 2–3.5 mm of posterior movement. Therefore, patients presenting at least 2 mm of planned posterior mesialization were included as representing moderate anchorage conditions. This operational threshold (≥ 2 mm) represents the lower bound of the classical 1/4–1/2 extraction-space range described in standard orthodontic biomechanics, ensuring that only cases with a clinically meaningful amount of posterior mesialization were included for analysis.

The inclusion criteria were as follows: (1) bilateral extraction of the maxillary and mandibular first premolars; (2) moderate anchorage requirement, defined as ≥ 2 mm of mesial movement of the posterior teeth; (3) treatment with Invisalign^®^ (Align Technology, San José, CA, USA); (4) completion of the initial aligner series without midcourse refinement; (5) full permanent dentition excluding third molars; and (6) availability of complete digital dental models obtained before and after treatment.

All cases were consecutively collected from the same clinical database to minimize selection bias. The sample included both adolescents and adults, representing the typical age distribution of patients requiring moderate anchorage in clinical practice.

### Digital model superimposition and measurement

**Fig. 1:**
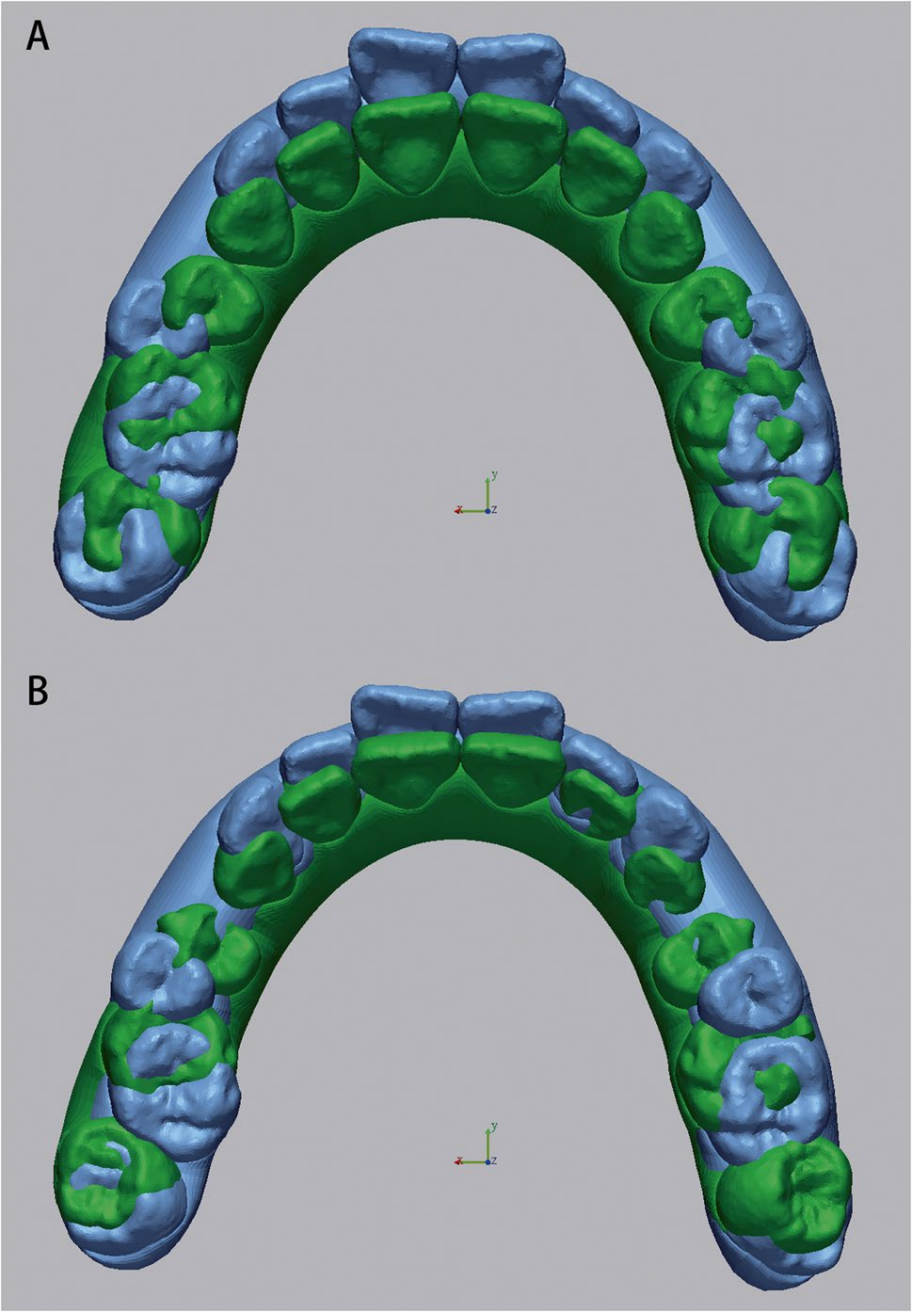
**A** Superimposition of pretreatment and planned models. **B** Superimposition of pretreatment and achieved models (Maxilla)

**Fig. 2:**
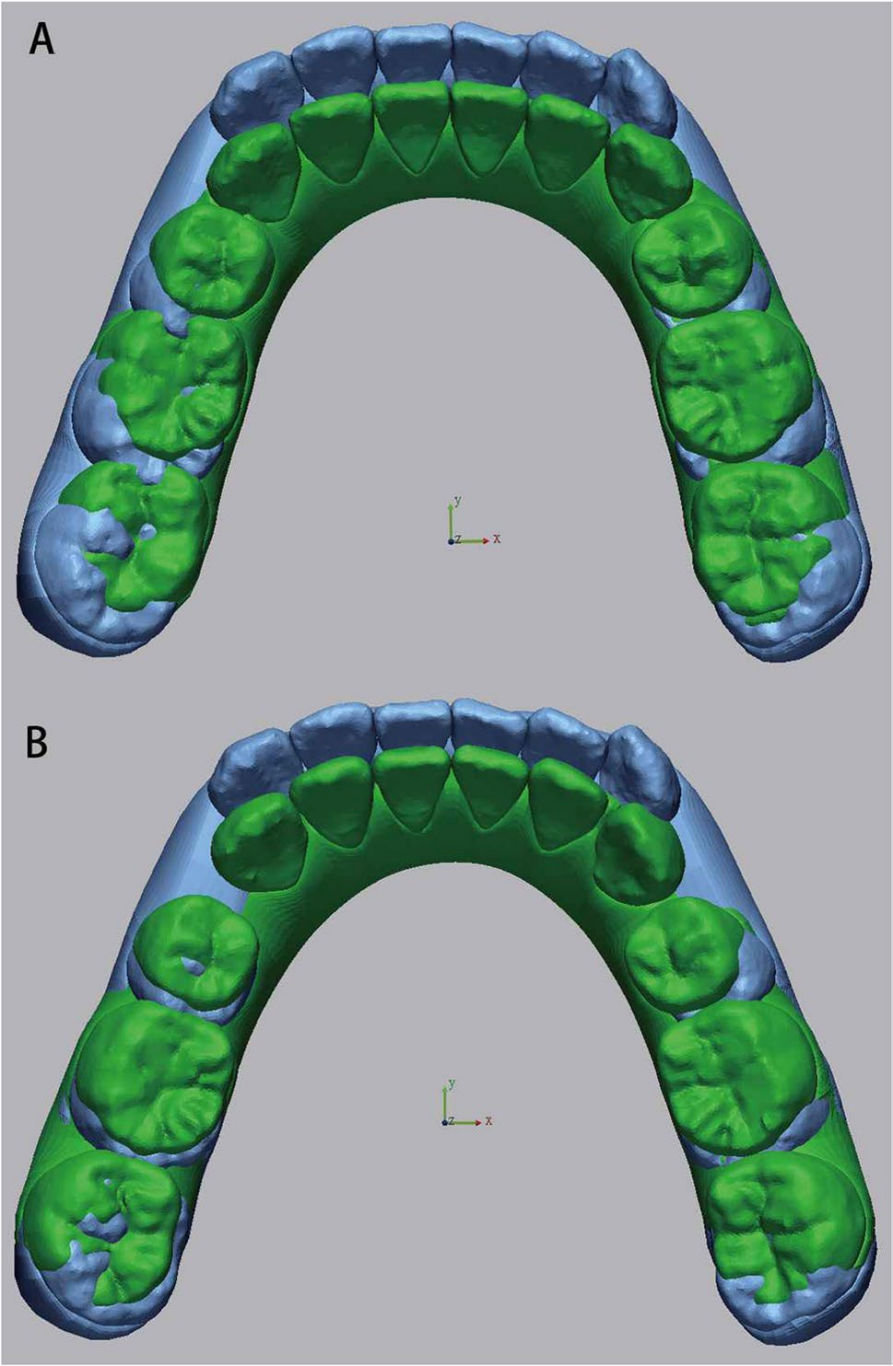
**A** Superimposition of pretreatment and planned models. **B** Superimposition of pretreatment and achieved models (Mandible)

Pre- and post-treatment intraoral scans were performed using the iTero intraoral scanner (Align Technology, San José, CA, USA). Virtual dental models at both timepoints were exported from the ClinCheck^®^ software (Align Technology Inc.) and subsequently imported into Geomagic Control X software (3D Systems, Rock Hill, NC, USA) for superimposition. For maxillary models, the stable palatal vault region encompassing the third palatal rugae was used as the reference area, which is morphologically stable during orthodontic treatment [26, 27]. For mandibular models, because no rigid anatomical reference exists, a surface-based best-fit registration was adopted following the validated workflow of Chan et al. [28] and Meade et al. [29]. Minor scanning artifacts were trimmed prior to alignment. A two-step protocol was performed: first, an initial alignment established rough positioning, followed by an best-fit registration with 50 iterations and 80% surface sampling. This workflow has been validated in previous studies(28, 29) to provide high accuracy and excellent reproducibility for digital model alignment, enabling precise superimposition without relying on manually defined anatomical landmarks (Figs. 1 and 2).

**Fig. 3:**
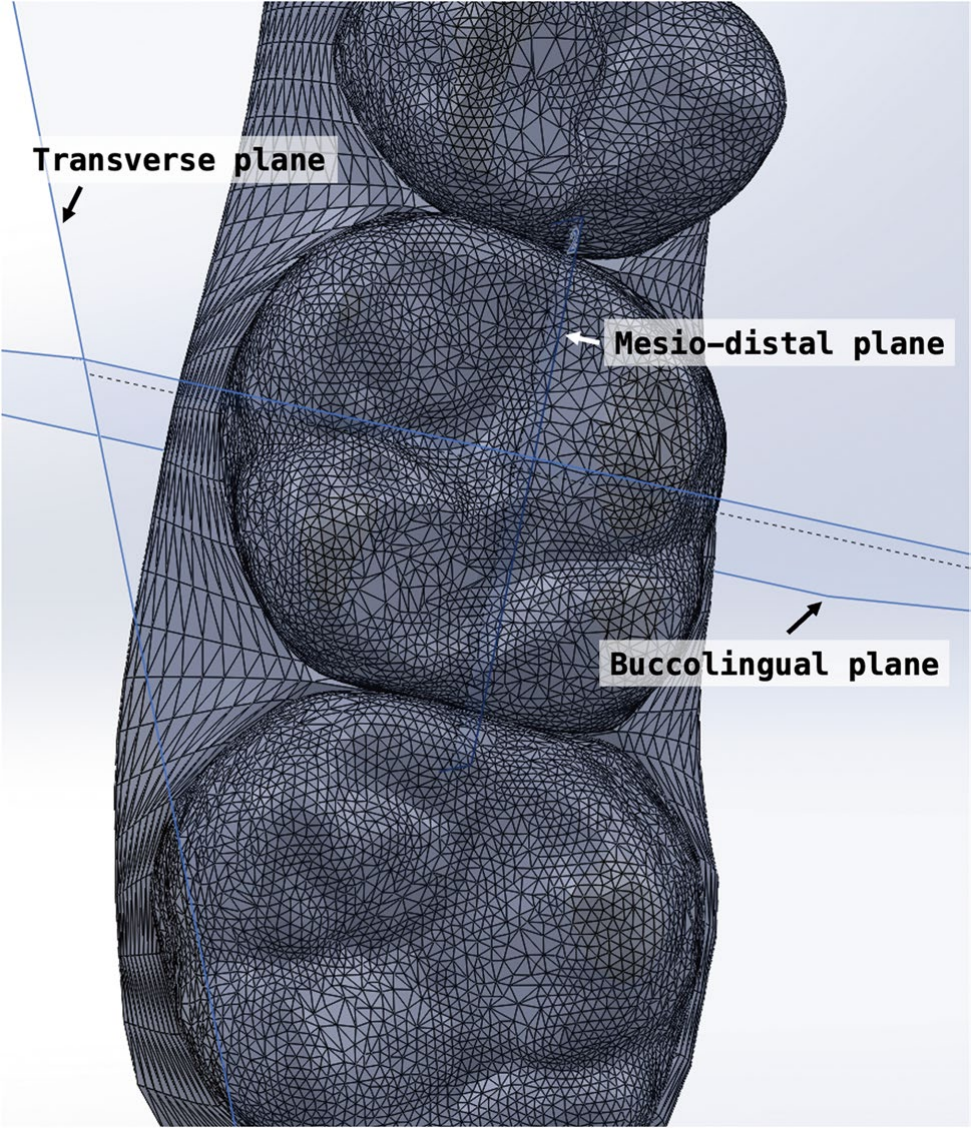
Three-dimensional coordinate system

Following superimposition, a three-dimensional (3D) coordinate system was established to quantify tooth movement. Reference planes were defined using the pretreatment model. The transverse plane was constructed using the mesial cusps of the bilateral first molars and the mesioincisal edge of the right maxillary central incisor (Fig. 3). The midsagittal plane was defined as perpendicular to the transverse plane, passing through the gingival zeniths of the maxillary central incisors. The linear and angular measurements were performed according to those studies by Ren et al [19] and Wen et al. [30].

Mesiodistal linear measurements were obtained by identifying the buccal cusp tips of the second premolars and the mesio-buccal cusp tips of the first molars. Sagittal displacement was calculated as the difference in mesiodistal position between the post-treatment and pre-treatment models, measured along the sagittal axis established from the reference coordinate system. Mesial movement was defined as positive.

**Fig. 4:**
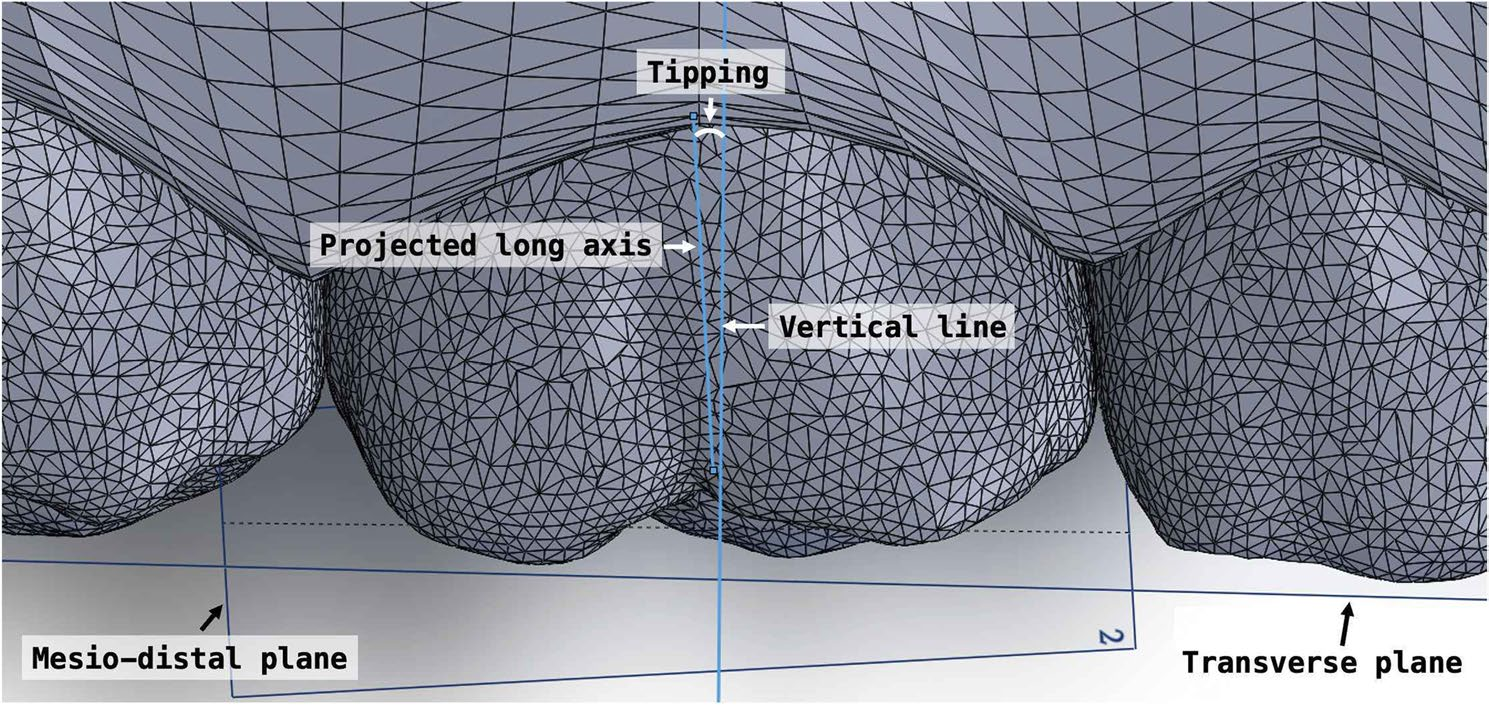
Measurement of tipping

Crown angulation was evaluated in three dimensions: tipping, rotation, and buccolingual inclination. To assess mesiodistal tipping, the most mesial and distal points along the central occlusal groove of each tooth were identified. These points and their projections onto the transverse plane defined the individual mesiodistal plane of the tooth. The long axis of the molar crown was constructed by connecting the most occlusal and gingival points along the buccal groove. This axis was projected onto the mesiodistal plane, and the angle formed between this projected line and a vertical line perpendicular to the transverse plane was defined as the mesiodistal tipping angle (Fig. 4).

**Fig. 5:**
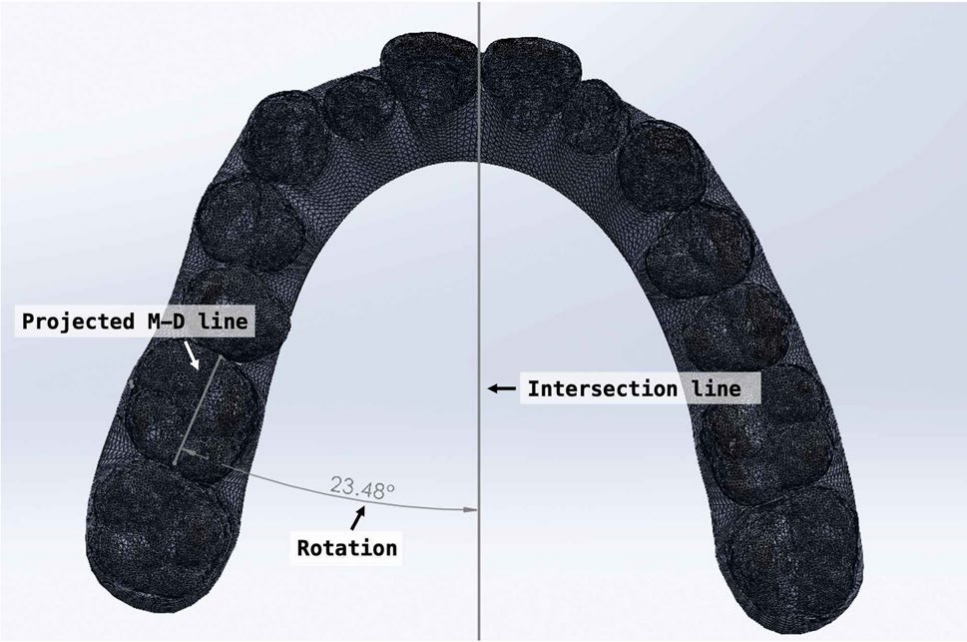
Measurement of rotation

For rotation measurements, the mesial and distal edge points of the occlusal surface were projected onto the transverse plane. The angle between the resulting line and the midsagittal plane was defined as the absolute rotation angle (Fig. 5).

**Fig. 6:**
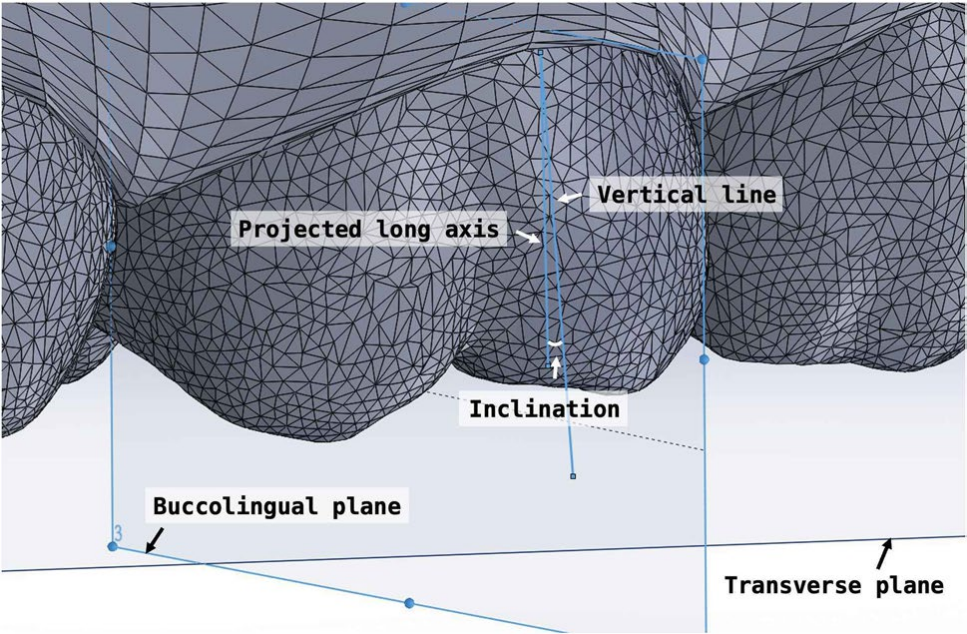
Measurement of inclination

Buccolingual inclination was assessed by projecting the crown axis onto the buccolingual plane, which was defined as perpendicular to both the transverse and mesiodistal planes. The angle between this projected axis and the line perpendicular to the transverse plane represented the buccolingual inclination (Fig. 6).

## Attachment design groups

**Fig. 7:**
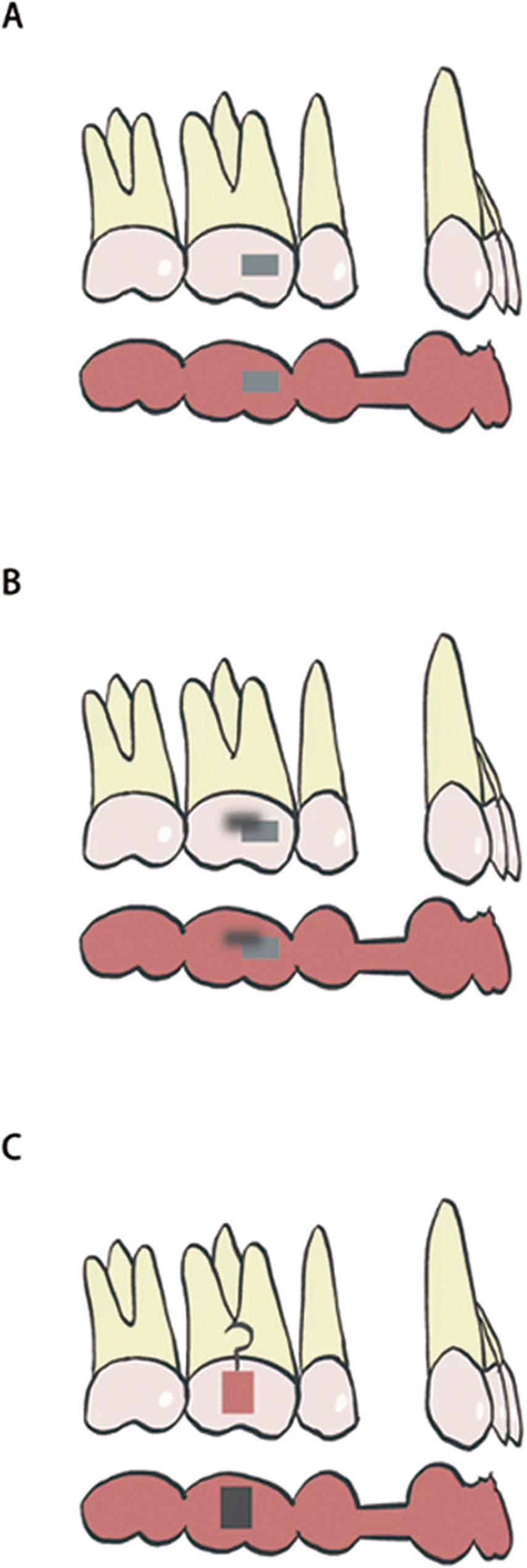
Illustration of the attachment and power arm configurations on the first molar. **A** Single rectangular attachment; **B** Double rectangular attachment; **C** Power arm

To evaluate the influence of attachment design on molar angulation, the maxillary and mandibular first molars were categorized into three subgroups based on the attachment used during treatment: (1) single rectangular attachment group, (2) power arm group, and (3) double rectangular attachment group (Fig. 7). This classification enabled comparison of the effectiveness of different attachment types in controlling mesial tipping of the first molars. Each patient received one consistent attachment type to avoid intra-patient variability.

### Statistical analysis

All statistical analyses were conducted using SPSS software (version 19.0; IBM Corp., Armonk, NY, USA). All measurements were performed independently by a single calibrated examiner and repeated after a two-week interval to assess intraexaminer reliability. The consistency of repeated measurements was evaluated using Pearson correlation coefficients and Bland-Altman plots.

Normality of data distribution was confirmed prior to analysis. Paired t-tests were used to compare predicted and achieved tooth movements. A significance level of *P* < 0.05 was considered statistically significant. One-way analysis of variance (ANOVA) was employed to assess differences in mesiodistal tipping of the first molars among the three attachment groups. When the ANOVA indicated a significant difference (*P* < 0.05), post hoc comparisons were conducted using the Tukey honestly significant difference (HSD) test.

Linear regression analysis was performed to evaluate the influence of patient-specific variables, including age, sex, and vertical skeletal pattern, on the discrepancy between predicted and actual tooth movements. A significance threshold of *P* < 0.05 was applied.

A priori power analysis was conducted based on data from a previous study [21], using mesial movement of the posterior teeth as the primary outcome variable. With a significance level of 0.05 and a desired power of 90%, the minimum sample size required was calculated to be 30 participants.

## Results

### Patient characteristics

The characteristics of included patients are shown in Table 1. This study included 32 patients (24 females and 8 males; mean age 23.9 ± 6.4 years) with total 194 posterior teeth fulfilled the eligibility criteria. Among them, 10 adolescents and 22 adults participated in this study, averaging 23.97 ± 6.36 years old. With respect to vertical skeletal pattern, 13 patients were classified as hyperdivergent, 11 as normodivergent and 8 as hypodivergent.

**Table 1:** Characteristics of included patients

Characteristics	Mean ± SD/*N* (%)
Sex	
Female	24 (75%)
Male	8 (25%)
Age	23.97 ± 6.36
Adolescent	10 (31.3%)
Adult	22 (68.7%)
Vertical skeletal pattern	
Hyperdivergent	13 (40.6%)
Normodivergent	11 (34.4%)
Hypodivergent	8 (25%)

### Differences between planned and achieved tooth movements

**Fig. 8:**
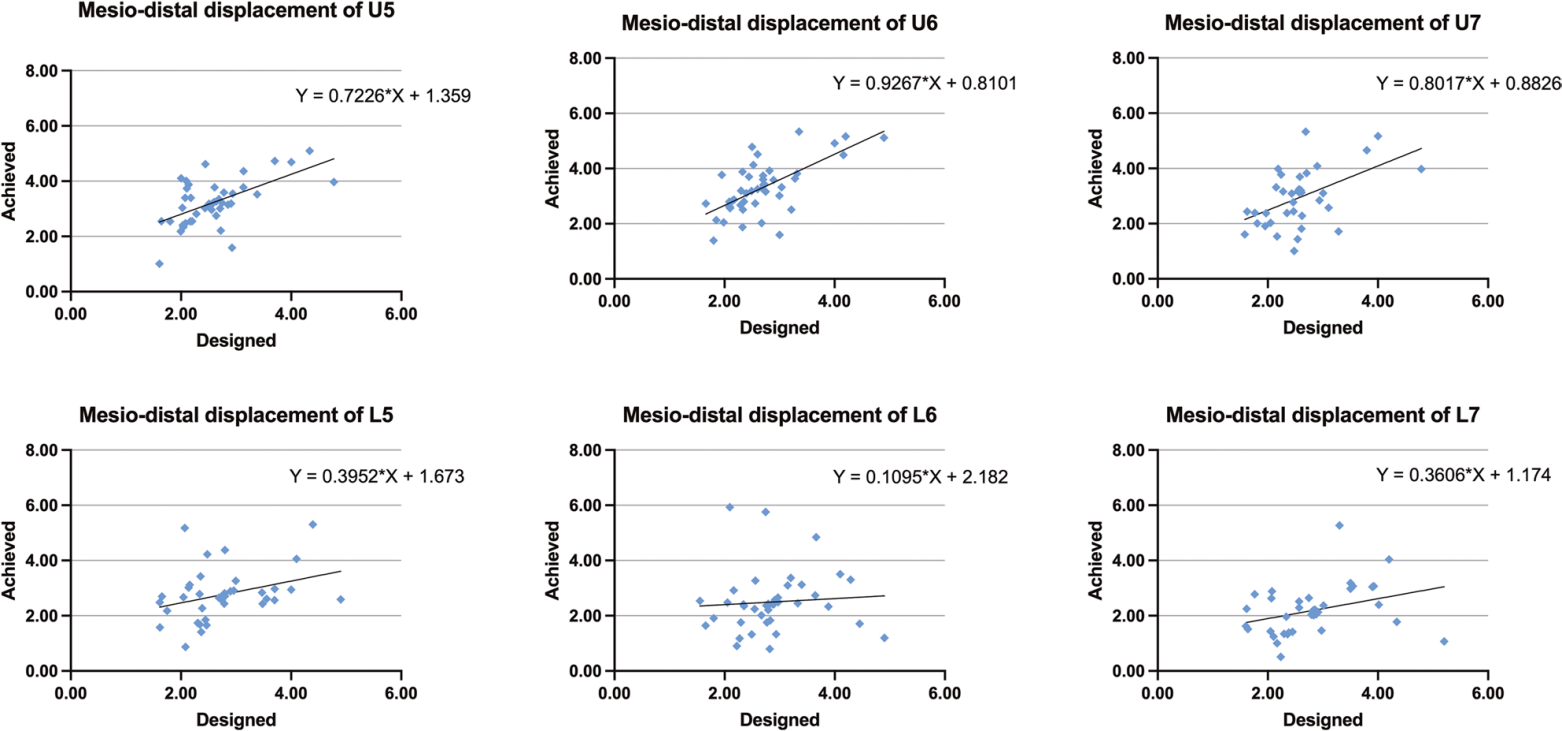
Scatter plots of predicted vs. achieved mesial movement distances for posterior teeth

**Fig. 9:**
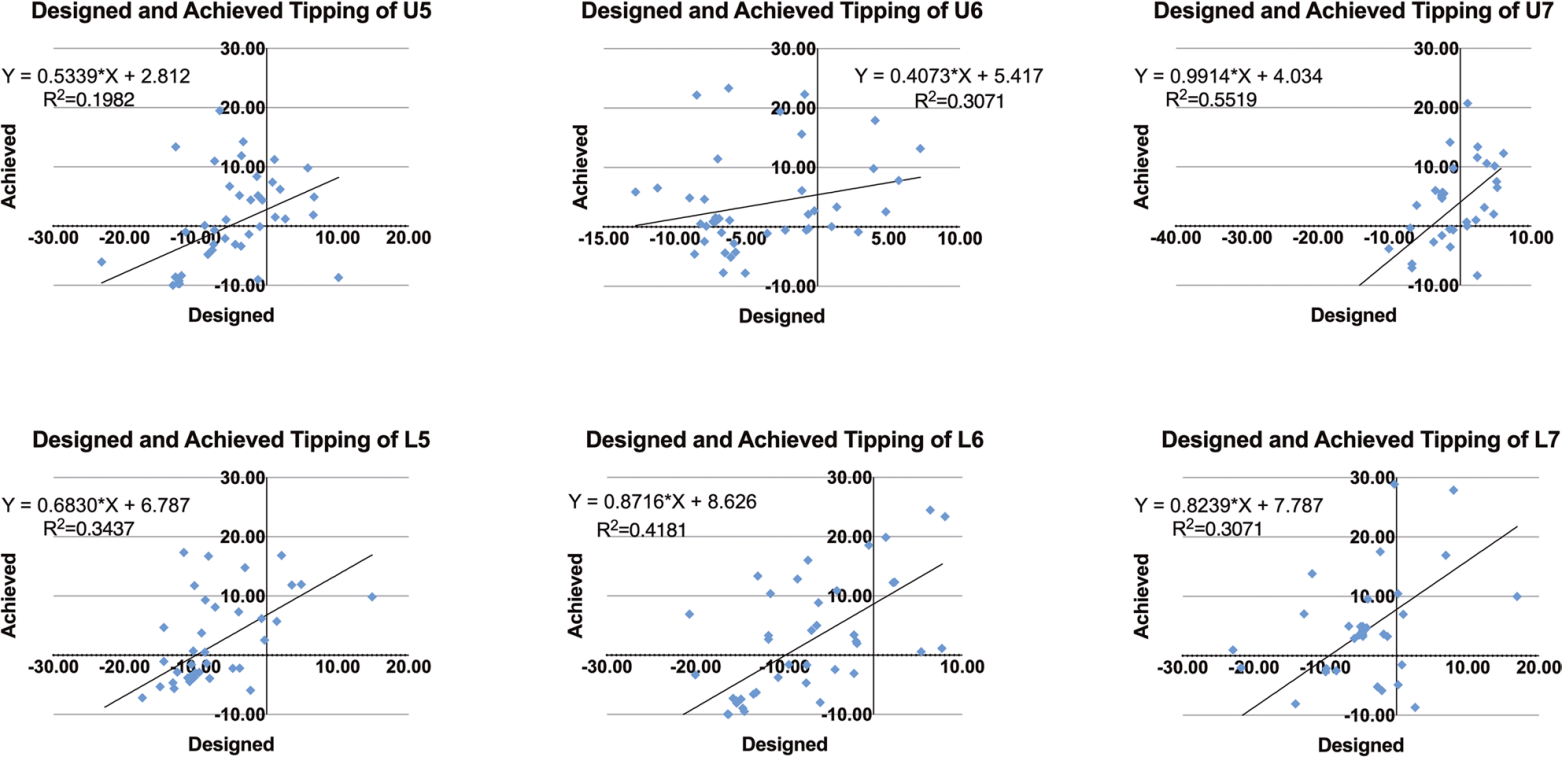
Scatter plots of predicted vs. achieved tipping for posterior teeth

Tables 2 (maxilla) and 3 (mandible) summarize the predicted and achieved values for mesiodistal displacement, inclination, rotation, and crown tipping of the second premolars, first molars, and second molars in both the maxillary and mandibular arches. Figs. 8 and 9 also illustrate the scatter plots of comparing the predicted and achieved displacement and tipping across all posterior teeth.

**Table 2:** Designed and achieved changes in distance, inclination, rotation and tipping of maxillary posterior teeth

Movement	Designed	Achieved	Difference	*P*
Second premolar				
Displacement	2.60 ± 0.65	3.24 ± 0.81	0.64 ± 0.68	< 0.001
Inclination	0.16 ± 4.54	-1.25 ± 6.04	-1.41 ± 5.16	0.073
Rotation	4.60 ± 7.48	5.05 ± 7.79	0.45 ± 0.16	0.625
Tipping	-5.45 ± 6.77	-0.10 ± 8.12	5.35 ± 7.93	< 0.001
First molar				
Displacement	2.67 ± 0.75	3.28 ± 0.94	0.61 ± 0.71	< 0.001
Inclination	-1.20 ± 6.15	-4.67 ± 5.34	-3.47 ± 5.68	< 0.001
Rotation	0.20 ± 5.76	2.65 ± 6.47	2.63 ± 4.93	0.001
Tipping	-4.08 ± 4.75	3.75 ± 7.87	7.84 ± 8.14	< 0.001
Second molar				
Displacement	2.57 ± 0.60	2.94 ± 0.96	0.37 ± 0.84	0.007
Inclination	1.24 ± 6.11	-2.64 ± 6.14	-3.89 ± 7.20	< 0.001
Rotation	1.23 ± 6.30	1.29 ± 6.33	0.05 ± 5.45	0.951
Tipping	-2.49 ± 6.93	1.56 ± 9.25	4.06 ± 6.19	< 0.001

Displacement: +, mesial displacement of posterior teeth; -, distal displacement of posterior teeth. Inclination: +, lingual inclination; -, buccal inclination. Rotation: +, mesial-lingual rotation; -, distal-lingual rotation. Tipping: +, mesial tipping; -, distal tipping

**Table 3:** Designed and achieved changes in distance, inclination, rotation and tipping of mandibular posterior teeth

Movement	Designed	Achieved	Difference	*P*
Second premolar				
Displacement	2.76 ± 0.75	2.76 ± 0.89	0.004 ± 0.96	0.978
Inclination	-0.91 ± 7.66	-2.50 ± 6.87	-1.59 ± 6.59	0.131
Rotation	3.80 ± 12.93	9.77 ± 8.72	5.97 ± 8.81	< 0.001
Tipping	-7.89 ± 6.83	1.39 ± 7.95	9.29 ± 6.80	< 0.001
First molar				
Displacement	2.87 ± 0.72	2.50 ± 1.07	-0.38 ± 1.25	0.057
Inclination	-0.59 ± 8.00	-0.16 ± 7.50	0.44 ± 6.16	0.647
Rotation	0.39 ± 5.41	4.23 ± 5.74	3.91 ± 6.11	< 0.001
Tipping	-7.97 ± 7.85	1.68 ± 10.58	9.65 ± 8.14	< 0.001
Second molar				
Displacement	2.82 ± 0.80	2.19 ± 0.87	-0.63 ± 0.97	< 0.001
Inclination	-3.00 ± 8.89	-3.39 ± 5.54	-0.39 ± 8.73	0.776
Rotation	0.07 ± 5.63	1.83 ± 5.72	1.77 ± 6.13	0.076
Tipping	-4.76 ± 7.79	3.86 ± 11.58	8.63 ± 9.74	< 0.001

Displacement: +, mesial displacement of posterior teeth; -, distal displacement of posterior teeth. Inclination: +, lingual inclination; -, buccal inclination. Rotation: +, mesial-lingual rotation; -, distal-lingual rotation. Tipping: +, mesial tipping; -, distal tipping

### Maxillary arch

For the maxillary second premolars, statistically significant differences were observed between the predicted and achieved values for mesiodistal displacement and crown tipping (*P* < 0.001). The achieved displacement was greater than predicted, accompanied by increased mesial tipping. No significant differences were found for inclination or rotation (*P* > 0.05).

For the maxillary first molars, all measured parameters showed significant differences between the predicted and achieved values. The achieved mesial displacement was greater than the planned amount (*P* < 0.001), and the crowns exhibited significant mesial tipping (*P* < 0.001) and mesial-lingual rotation (*P* = 0.001). A significant difference was also detected in inclination (*P* < 0.001).

For the maxillary second molars, significant differences were observed in mesiodistal displacement (*P* = 0.007), tipping (*P* < 0.001), and inclination (*P* < 0.001), while rotation showed no significant difference (*P* = 0.951). The amount of achieved mesial movement was greater than predicted, with accompanying mesial tipping.

### Mandibular arch

For the mandibular second premolars, significant differences were observed in crown tipping (*P* < 0.001) and rotation (*P* < 0.001), whereas mesiodistal displacement and inclination showed no significant differences (*P* > 0.05).

For the mandibular first molars, significant differences were found in crown tipping (*P* < 0.001) and rotation (*P* < 0.001). No significant differences were detected in mesiodistal displacement (*P* = 0.057) or inclination (*P* = 0.647).

For the mandibular second molars, significant differences were observed in mesiodistal displacement (*P* < 0.001) and tipping (*P* < 0.001), while inclination and rotation showed no significant differences (*P* > 0.05). The achieved mesial movement was smaller than predicted.

To provide a clearer overview of the overall outcomes, a summary table (Table 4) was added to synthesize which tooth movements showed statistically significant discrepancies between predicted and achieved positions in each arch. Table 4 complemented the detailed results presented in Tables 2 and 3 and facilitates a concise visualization of the main findings.

**Table 4:** Summary of significant discrepancies between predicted and achieved tooth movements

Arch	Tooth	Mesial Displacement	Mesial Tipping	Rotation	Buccolingual Inclination
Maxilla	2nd premolar	**✓**	**✓﻿**	**✓**	**-**
Maxilla	1st molar	**✓**	**✓**	**✓**	**✓**
Maxilla	2nd molar	**✓﻿**	**✓**	**-**	**✓**
Mandible	2nd premolar	**✓**	**✓**	**✓**	**-**
Mandible	1st molar	**-**	**✓**	**-**	**✓**
Mandible	2nd molar	**✓**	**✓**	**-**	**-**

✓ = significant discrepancy (*P* < 0.05)

- = no significant difference

### Effect of attachment design

To evaluate the influence of attachment design on first molar tipping, both maxillary and mandibular first molars were divided into three groups based on attachment configuration: single rectangular attachment group, power arm group, and double rectangular attachment group.

**Fig. 10:**
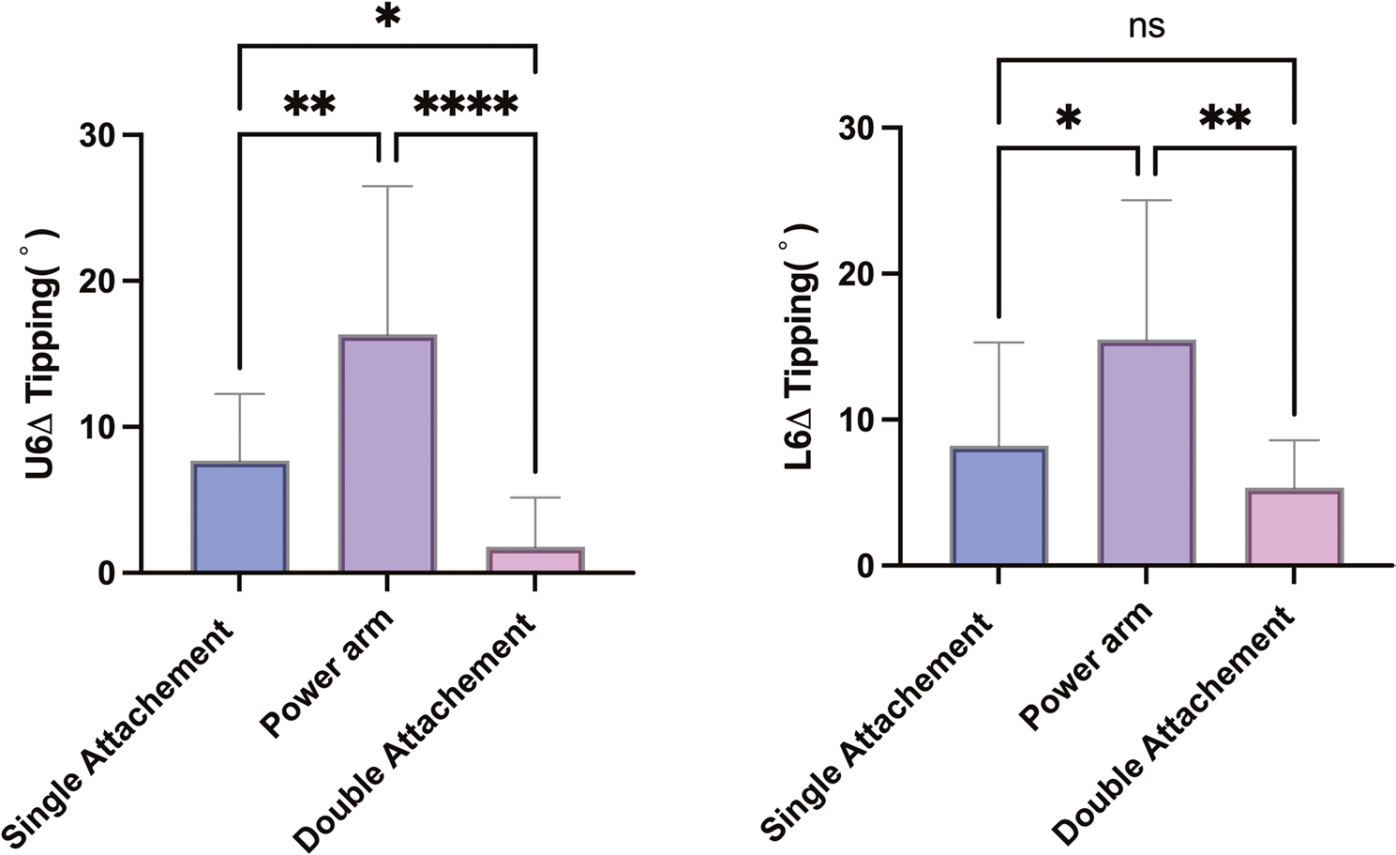
Comparison of mesial tipping of the maxillary and mandibular first molars among three different attachment designs

Significant differences in mesiodistal tipping were observed among the three groups in both the maxillary and mandibular arches (*P* < 0.05). In the maxilla, the power arm group demonstrated the greatest degree of tipping, significantly higher than the single attachment group (*P* < 0.05). The double attachment group exhibited the least amount of tipping, significantly lower than both the power arm and single attachment groups (*P* < 0.05). In the mandible, a similar pattern was observed: the power arm group showed the largest tipping, significantly greater than the single attachment group, while the double attachment group again demonstrated the smallest tipping, significantly lower than the power arm group (*P* < 0.05) (Fig. 10).

### Factors associated with tooth movement accuracy

Multiple linear regression analysis was conducted to assess the influence of age, sex, and vertical skeletal pattern on the discrepancy between predicted and achieved mesial movement (Tables 5, 6 and 7). Regression analysis confirmed that age was not a significant predictor of discrepancies between predicted and achieved movement. No significant associations were found for sex. However, vertical skeletal pattern was significantly associated with certain tooth movement discrepancies in both arches.

**Table 5:** Linear mixed-effect model analyses for differences between designed and achieved tooth movement of first molar

	Maxilla		Mandible	
Predictor	*B*(95%CI)	*P*	*B*(95%CI)	*P*
Displacement				
Age	0.033 (-0.01, 0.07)	0.06	-0.063 (-0.13, 0.01)	0.056
Sex (Female)	-0.223 (-0.65, 0.21)	0.299	0.626 (-0.35, 1.61)	0.203
Vertical skeletal pattern				
Hyperdivergent	-0.397 (-0.86, 0.62)	0.088	0.296 (-0.58, 1.18)	0.5
Normodivergent	Referent		Referent	
Hypodivergent	-0.958 (-1.45, -0.46)	0.001	-0.082 (-1.17, 1.01)	0.879
Inclination				
Age	-0.04 (-0.35, 0.27)	0.796	0.15 (-0.18, 0.48)	0.355
Sex (Female)	1.410 (-2.49, 5.32)	0.47	3.203 (-1.71, 8.11)	0.195
Vertical skeletal pattern				
Hyperdivergent	-3.41 (-7.59, 0.77)	0.107	-0.385 (-4.79, 4.03)	0.861
Normodivergent	Referent		Referent	
Hypodivergent	0.632 (-3.85, 5.11)	0.777	2.019 (-3.45, 7.49)	0.459
Rotation				
Age	-0.259 (-0.51, -0.01)	0.045	-0.271 (-0.59, 0.05)	0.091
Sex (Female)	1.542 (-1.66, 4.74)	0.974	0.404 (-4.38, 5.2)	0.865
Vertical skeletal pattern				
Hyperdivergent	2.99 (-0.43, 6.41)	0.085	0.291 (-4.0, 4.58)	0.892
Normodivergent	Referent		Referent	
Hypodivergent	-0.009 (-3.68, 3.66)	0.996	-3.551 (-8.87, 1.77)	0.185
Tipping				
Age	-2.326 (-7.57, 2.92)	0.376	0.291 (-0.12, 0.7)	0.158
Sex (Female)	0.307 (-0.11, 0.72)	0.143	0.26 (-5.92, 6.44)	0.932
Vertical skeletal pattern				
Hyperdivergent	-5.645 (-11.26, -0.34)	0.049	-0.896	0.745
Normodivergent	Referent		Referent	
Hypodivergent	-7.836 (-13.85, -1.82)	0.012	-8.821 (-15.70, -1.95)	0.013

**Table 6:** Linear mixed-effect model analyses for differences between designed and achieved tooth movement of second premolar

	Maxilla		Mandible	
Predictor	*B*(95%CI)	*P*	*B*(95%CI)	*P*
Displacement				
Age	0.024 (-0.01, 0.06)	0.159	-0.056 (-0.10, -0.01)	0.022
Sex (Female)	-0.138 (-0.56, 0.28)	0.512	0.491 (-0.25, 1.23)	0.188
Vertical skeletal pattern				
Hyperdivergent	-0.177 (-0.63, 0.27)	0.431	0.322 (-0.32, 0.97)	0.318
Normodivergent	Referent		Referent	
Hypodivergent	-0.876 (-1.36, -0.39)	0.001	-0.314 (-1.11, 0.49)	0.431
Inclination				
Age	0.169 (-0.12, 4.56)	0.238	0.177 (-0.28, 0.64)	0.438
Sex (Female)	0.995 (-2.61, 4.61)	0.581	-5.82 (-14.07, 2.43)	0.159
Vertical skeletal pattern				
Hyperdivergent	-1.834 (-5.69, 2.03)	0.343	-0.996 (-4.57, 2.85)	0.492
Normodivergent	Referent		Referent	
Hypodivergent	-0.795 (-4.94, 3.35)	0.70	-1.338 (-6.65, 3.98)	0.351
Rotation				
Age	0.188 (-0.15, 0.53)	0.27	-0.217 (-0.70, 0.27)	0.373
Sex (Female)	-1.479 (-5.79, 2.83)	0.491	-1.054 (-8.67, 6.56)	0.781
Vertical skeletal pattern				
Hyperdivergent	-1.052 (-5.66, 3.55)	0.647	-1.271 (-7.89, 5.35)	0.699
Normodivergent	Referent		Referent	
Hypodivergent	0.94 (-3.99, 5.88)	0.702	-0.368 (-8.56, 7.83)	0.928
Tipping				
Age	-0.007 (-0.43, 0.44)	0.975	0.191 (-0.16, 0.55)	0.283
Sex (Female)	-2.994 (-8.76, 2.48)	0.275	-0.24 (-5.79, 5.31)	0.931
Vertical skeletal pattern				
Hyperdivergent	-2.52 (-8.37, 3.33)	0.389	-4.219 (-9.05, 0.61)	0.85
Normodivergent	Referent		Referent	
Hypodivergent	-4.101 (-10.37, 2.17)	0.194	-5.87 (-11.85, 0.11)	0.054

**Table 7:** Linear mixed-effect model analyses for differences between designed and achieved tooth movement of second molar

	Maxilla		Mandible	
Predictor	*B*(95%CI)	*P*	*B*(95%CI)	*P*
Displacement				
Age	0.012 (-0.04, 0.06)	0.635	-0.020 (-0.07,0.03)	0.448
Sex (Female)	0.055 (-0.55, 0.66)	0.853	0.223 (-0.55, 0.99)	0.56
Vertical skeletal pattern				
Hyperdivergent	-0.264 (-0.91, 0.38)	0.413	0.826 (0.13, 1.52)	0.021
Normodivergent	Referent		Referent	
Hypodivergent	-0.749 (-1.47, -0.30)	0.042	0.51 (-0.33, 1.35)	0.227
Inclination				
Age	0.177 (-0.26, 0.62)	0.423	0.102 (-0.4, 0.61)	0.683
Sex (Female)	1.828 (-3.37, 7.03)	0.481	3.362 (-397, 10.69)	0.358
Vertical skeletal pattern				
Hyperdivergent	-4.899 (-10.50, 0.70)	0.084	-2.199 (-8.82, 4.42)	0.505
Normodivergent	Referent		Referent	
Hypodivergent	-1.974 (-8.19, 4.25)	0.524	3.511 (-4.52, 11.54)	0.381
Rotation				
Age	-0.018 (-0.36, 0.33)	0.916	-0.261 (-0.61,0.08)	0.135
Sex (Female)	0.781 (-3.29, 4.86)	0.700	1.415(-3.64, 6.47)	0.573
Vertical skeletal pattern				
Hyperdivergent	-2.043 (-6.43, 2.35)	0.352	-1.01 (-5.57, 3.56)	0.656
Normodivergent	Referent		Referent	
Hypodivergent	-0.733 (-5.61, 4.15)	0.762	-4.307 (-9.84, 1.23)	0.123
Tipping				
Age	0.199 (-0.18, 0.58)	0.298	0.491 (0.01, 0.98)	0.048
Sex (Female)	-3.423 (-7.89, 1.05)	0.129	-5.791 (-12.89, 1.31)	0.107
Vertical skeletal pattern				
Hyperdivergent	1.722 (-3.09, 6.54)	0.473	-1.346 (-7.75, 5.06)	0.672
Normodivergent	Referent		Referent	
Hypodivergent	-1.441 (-6.79, 3.91)	0.588	-11.203 (-18.97, 3.43)	0.006

In the maxillary arch, patients with low-angle skeletal patterns exhibited significantly greater discrepancies in molar tipping for both the first and second molars. Conversely, high-angle patients demonstrated significantly larger discrepancies in inclination for the second premolars and first molars. No significant associations were found for mesiodistal displacement or rotation in relation to vertical skeletal pattern.

In the mandibular arch, similar trends were observed. Low-angle patients showed significantly greater tipping discrepancies in the first and second molars. High-angle individuals exhibited greater inclination discrepancies in the first molars, consistent with the findings in the maxilla. No significant associations were identified for rotation or mesiodistal displacement, except for a significant relationship between vertical pattern and displacement in the second molars.

## Discussion

Clear aligner therapy has evolved incredibly over the years, becoming increasingly widely accepted by orthodontists and patients. However, challenges remain, especially in extraction cases where posterior teeth need to mesialize. In these situations, mesial tipping of the posterior teeth is a common side effect. Several factors contribute to this phenomenon: first, the inherent mesial angulation of the molars or premolars, which predisposes them to tipping during movement; second, the forward occlusal force between the upper and lower arches, which causes both the upper and lower molars to tip mesially as they move in relation to one another; last but not least, the insufficient anchorage provided by the aligner system, which may fail to counteract these tipping forces. Simply relying on the aligners cannot be enough to prevent posterior teeth from mesial tipping. Accordingly, the present findings provide new perspectives on how biomechanical differences between the maxillary and mandibular arches influence posterior mesialization with clear aligners. These insights highlight key factors (sex, age or vertical skeletal patterns) that may interfere with controlled tooth movement. Moreover, optimizing attachment configuration appears to enhance both the efficacy and predictability of bodily mesialization, offering practical guidance for improving treatment outcomes in moderate anchorage cases.

Previous studies [13, 19–21] have reported a relatively low accuracy in achieving mesial movement of posterior teeth with clear aligner therapy. However, in moderate anchorage extraction cases, such posterior tooth movement is often clinically necessary and cannot be avoided. Unlike most prior research that has predominantly focused on maximum anchorage conditions, this study specifically examined cases characterized by ≥ 2 mm of planned posterior tooth movement under moderate anchorage control. The objective was not only to assess the actual versus predicted tooth movement outcomes, but also to investigate the influence of patient-related and biomechanical factors on the efficacy of space closure. Besides, mesial tipping of first molar was compared by three groups of different attachment designed.

The results of this study revealed significant discrepancies between the predicted and actual mesial movement of all included posterior teeth in both the maxillary and mandibular arches. Specifically, all posterior teeth exhibited notable mesial tipping during mesialization, rather than demonstrating controlled bodily movement. As previously highlighted by Robertson et al. [18] clear aligner therapy often falls short in achieving simultaneous crown and root control during complex tooth movements, such as posterior mesialization. The current findings reinforce this limitation and suggest that mesial tipping remains a common biomechanical challenge when managing extraction cases under moderate anchorage with clear aligners.

This tendency was particularly pronounced in the maxillary arch, where the first molars demonstrated greater-than-anticipated mesial displacement accompanied by significant mesial tipping. These findings are consistent with those of previous studies by Dai et al. [20, 21] and Ren et al. [19] which similarly reported excessive mesial movement of maxillary posterior teeth during space closure with strong anchorage using clear aligners. Such results suggest that even under varying anchorage conditions, the maxillary posterior segment remains prone to anchorage loss. Relying solely on appliance retention and aligner fit may therefore be insufficient to ensure adequate posterior anchorage control in clinical practice. The anchorage loss of the maxilla could be explained by following biomechanical and anatomical factors. First, the maxilla generally exhibits lower bone density and thinner cortical plates than the mandible [31] These factors would reduce resistance to mesial displacement and may facilitate uncontrolled tipping under similar force systems. Second, the trifurcated root morphology of the maxillary first molar creates a center of resistance with greater individual variation, making controlled root movement more difficult [32] If the line of force does not pass through the center of resistance, tipping rather than bodily movement is more likely to occur. Third, although the maxilla offers less resistance, localized areas such as the zygomatic buttress show denser cortical bone, particularly adjacent to the mesio-buccal root. Increasing the possibility of altering the path of movement and contribute to uncontrolled tooth movement.

In contrast, the discrepancy in movement was smaller than that observed in the maxillary arch, suggesting more effective anchorage control in the mandible. This finding aligns with results typically seen in fixed appliance therapy. Interestingly, the actual mesial movement of the mandibular first and second molars was less than the planned amount, which differs from the findings of Dai et al. [20]. One possible explanation is that when the planned mesial movement exceeds 2 mm, the higher bone density in the mandible may hinder the full expression of tooth movement [33]. The overall reduced mesialization in the mandible compared to the maxilla may also be attributed to anatomical and biomechanical factors. Maxillary molars are generally more responsive to mesial forces, and the maxillary anterior segment contains larger teeth, potentially increasing the anchorage demand and facilitating molar movement. Despite the limited displacement, mandibular first molars still exhibited substantial mesial tipping, indicating that the observed movement may have occurred primarily through uncontrolled crown tipping rather than bodily translation. From a clinical standpoint, this means that overtreatment of planned mesial movement and anchorage preparation may be required in the mandible to compensate for reduced movement efficiency.

In clear aligner therapy, when excessive mesial tipping of posterior teeth occurs, additional appliances or auxiliary techniques are often necessary to correct crown angulation. At present, segmented arch mechanics remain the most commonly employed method to address posterior mesial tipping. However, incorporating fixed appliance techniques may compromise the efficiency and continuity of aligner-based treatment, undermining the philosophy of fully bracket-free therapy. When temporary skeletal anchorage devices (TADs) are used in conjunction with clear aligners, they are typically placed in the posterior region. As such, they offer limited effectiveness in counteracting mesial tipping that develops during posterior mesialization. This limitation has also been noted by Ren et al. [19] who reported that TADs alone may not be sufficient to maintain molar anchorage in extraction cases treated with clear aligners.

Dai et al. [21] previously reported no significant differences in mesial tipping of the maxillary first molars among various attachment designs. Similarly, Vongtiang et al. [13] observed slightly greater molar tipping in the power arm group compared to the control group under maximum anchorage conditions. When comparing different attachment configurations, we found that the double rectangular attachments offered the best control over molar tipping, while the power arm design surprisingly produced the greatest amount of crown tipping. This pattern seems to reflect several biomechanical aspects of how forces are transferred through the aligner. In the double-attachment design, the larger contact surface between the aligner and the tooth likely increases retention and allows better transmission of counteracting moments that help limit mesial crown tipping. By contrast, the vertical projection of the power arm can locally deform the aligner material when under continuous functional stress. Such deformation may slightly shift the direction of force relative to the molar’s center of resistance, resulting in a tipping tendency rather than a true bodily movement. Moreover, the hook-shaped configuration of the power arm may induce inward pulling of the aligner, contributing to aligner disengagement and uncontrolled crown movement. These mechanical issues together could explain why the clinical behavior of the power arm group did not match its theoretical advantage. Similar findings have been described by Vongtiang et al. [13], who also reported more tipping in power-arm-assisted movements than with standard attachments. Collectively, improving aligner retention, such as by using double attachments, appears beneficial for enhancing bodily mesialization control under moderate anchorage conditions.

These findings suggest that attachment design plays a crucial role in controlling molar tipping. This supports the conclusions of Li et al. [22] who reported that rational attachment design can improve root angulation control. The differing outcomes from Dai et al. [21] may be due to variations in anchorage conditions, as their work focused mainly on maximum anchorage cases, while the present study examined moderate anchorage situations. As the distance of planned mesial movement increases, the risk of uncontrolled crown tipping appears to rise accordingly.

The maxillary and mandibular first molars, as well as the mandibular second premolars, exhibited greater mesiolingual crown rotation than predicted, a situation that has been identified as unfavorable in the context of extraction treatment [14]. This observation is consistent with the findings reported by Dai et al. [20] who also noted excessive rotational deviation during space closure with aligners. This could be explained by the fact that the force vector applied by the aligner does not pass precisely through the tooth’s center of resistance. A residual moment is generated, producing mesiolingual rotation instead of pure translation. Clinically, this highlights the need for adequate attachment geometry or overcorrection programming to prevent residual rotations during posterior mesialization. In addition, the maxillary first molars demonstrated increased buccal crown inclination whereas the mandibular first molars showed a tendency toward lingual inclination. These buccolingual inclination patterns mirror those commonly observed in conventional fixed appliance therapy, where maxillary molars tend to incline buccally and mandibular molars exhibit a lingual tipping tendency. This tendency may result from the combined influence of occlusal contacts, arch form curvature, and aligner elasticity, which can produce unequal force vectors on the buccal and lingual cusps during mesialization. Specifically, the upper molars often experience buccal displacement due to palatal force vector components, whereas the lower molars are constrained lingually by the denser cortical bone on the mandibular buccal wall. Clinically, it emphasizes the importance of torque overcorrection in digital setup to maintain proper buccolingual inclination during space closure.

The results of the mixed linear regression analysis indicated that only vertical skeletal pattern was found to be a relevant factor. The influence of vertical skeletal pattern on tooth movement with aligners appears to reflect differences in both musculoskeletal and anatomical characteristics. In low-angle patients, stronger masticatory muscles and thicker cortical bone generate higher resistance to tooth movement, making bodily translation more difficult to achieve. Under such conditions, the crown tends to tip more readily in the mesiodistal direction, as greater force is required to produce equivalent root movement. In contrast, high-angle patients exhibit weaker masticatory loading and thinner cortical plates, which reduce vertical anchorage and the ability to resist crown displacement. Because aligners primarily transmit force at the crown level, with limited counteracting torque for root control, the imbalance between crown and root forces makes the tooth more prone to buccolingual inclination rather than mesiodistal tipping. This direction-specific tendency may be further amplified by the narrower alveolar housing and reduced occlusal stability commonly observed in high-angle individuals. Although both mesiodistal and buccolingual discrepancies can occur during mesialization, the predominance of buccolingual changes in high-angle patients and mesiodistal tipping in low-angle patients suggests that skeletal morphology influences not only the amount but also the direction of uncontrolled movement. Clinically, these findings highlight the importance of incorporating skeletal morphology into digital treatment planning, especially when aiming for precise posterior control with clear aligners.

This study has several limitations that should be acknowledged. First, its retrospective design limits control over confounding factors such as patient compliance, attachment wear, and individual biological variation. These factors might have influenced the degree of tooth movement achieved with aligners. Second, the sample size was relatively small, particularly after dividing the cases into subgroups by attachment design and skeletal pattern. As a result, the statistical power for detecting subtle intergroup differences may have been reduced. Third, the analysis relied on digital model superimposition, which reflects crown-level rather than root-level movement. The absence of CBCT data prevented direct assessment of root behavior, so the study cannot distinguish true bodily movement from controlled tipping. Fourth, the inclusion of both adolescents and adults could introduce heterogeneity due to residual growth in the younger subgroup. However, regression analysis showed that age was not a significant predictor of mesialization accuracy in this sample. Future studies with age-stratified samples and longitudinal designs are warranted to further validate this observation. Finally, the findings should be interpreted within the limitations of the Invisalign system and the specific range of moderate anchorage movements examined. While the results highlight meaningful biomechanical trends, they should not be generalized to other aligner systems or different anchorage protocols. Nevertheless, the present findings may serve as a foundation for optimizing digital treatment planning and attachment design in moderate-anchorage cases. Future studies combining CBCT-based root analysis, patient-specific finite element modeling, and larger prospective cohorts could further elucidate the mechanics of posterior mesialization with clear aligners [34].

## Conclusion

Within the limitations of this retrospective analysis, clear aligner therapy under moderate anchorage conditions demonstrated measurable discrepancies between predicted and achieved posterior tooth movements, particularly in the maxillary arch. Molar mesial tipping remained a frequent biomechanical limitation, whereas the use of double rectangular attachments appeared to provide improved control of crown angulation compared with single attachment or power arm. Vertical skeletal pattern influenced the expression of tooth movement: low-angle patients tended to show greater mesiodistal tipping discrepancies, while high-angle patients exhibited more buccolingual inclination deviations. These observations may help refine digital treatment planning and attachment design in extraction cases requiring controlled posterior mesialization. Further prospective, CBCT-based studies with larger and age-stratified samples are warranted to validate and expand upon these findings.

## Data Availability

The datasets generated and analyzed during the current study are available from the corresponding author upon reasonable request.
